# Intratumoural microbiota: a new frontier in cancer development and therapy

**DOI:** 10.1038/s41392-023-01693-0

**Published:** 2024-01-10

**Authors:** Yaqi Cao, Hui Xia, Xueyun Tan, Chunwei Shi, Yanling Ma, Daquan Meng, Mengmeng Zhou, Zhilei Lv, Sufei Wang, Yang Jin

**Affiliations:** 1 Department of Respiratory and Critical Care Medicine, Hubei Province Clinical Research Center for Major Respiratory Diseases, Key Laboratory of Respiratory Diseases of National Health Commission, State Key Laboratory for Diagnosis and Treatment of Severe Zoonotic Infectious Diseases, Union Hospital, Tongji Medical College, Huazhong University of Science and Technology, Wuhan, Hubei 430022 China; 2 Hubei Province Engineering Research Center for Tumour-Targeted Biochemotherapy, MOE Key Laboratory of Biological Targeted Therapy, Union Hospital, Tongji Medical College, Huazhong University of Science and Technology, Wuhan, Hubei 430022 China; 3 Hubei Province Key Laboratory of Biological Targeted Therapy, Union Hospital, Tongji Medical College, Huazhong University of Science and Technology, Wuhan, Hubei 430022 China; 4 Department of Pathogen Biology, School of Basic Medicine, Tongji Medical College, Huazhong University of Science and Technology, Wuhan, Hubei 430022 China

**Keywords:** Tumour immunology, Cancer

## Abstract

Human microorganisms, including bacteria, fungi, and viruses, play key roles in several physiological and pathological processes. Some studies discovered that tumour tissues once considered sterile actually host a variety of microorganisms, which have been confirmed to be closely related to oncogenesis. The concept of intratumoural microbiota was subsequently proposed. Microbiota could colonise tumour tissues through mucosal destruction, adjacent tissue migration, and hematogenic invasion and affect the biological behaviour of tumours as an important part of the tumour microenvironment. Mechanistic studies have demonstrated that intratumoural microbiota potentially promote the initiation and progression of tumours by inducing genomic instability and mutations, affecting epigenetic modifications, promoting inflammation response, avoiding immune destruction, regulating metabolism, and activating invasion and metastasis. Since more comprehensive and profound insights about intratumoral microbiota are continuously emerging, new methods for the early diagnosis and prognostic assessment of cancer patients have been under examination. In addition, interventions based on intratumoural microbiota show great potential to open a new chapter in antitumour therapy, especially immunotherapy, although there are some inevitable challenges. Here, we aim to provide an extensive review of the concept, development history, potential sources, heterogeneity, and carcinogenic mechanisms of intratumoural microorganisms, explore the potential role of microorganisms in tumour prognosis, and discuss current antitumour treatment regimens that target intratumoural microorganisms and the research prospects and limitations in this field.

## Introduction

Approximately 38 trillion microorganisms are found in the human microbiota, including bacteria, fungi, and viruses, and their number roughly equals that of human cells.^1,2^ These microorganisms have previously been found in open cavities and organs such as the gut, skin, mouth, and vagina.^3^ However, with the breakthroughs of technology, the tissues and organs once considered sterile, including the lung, breast, liver, pancreas, prostate, and kidney, have also demonstrated to harbour low-biomass microbial communities, which leads to further research in related fields.^4,5^ In particular, the concept of intratumoural microbiota present in tumour tissues is proposed,^6^ and such microorganisms have been found in at least 33 major cancer types.^7–9^ The intratumoural microbiota is an integral part of the tumour microenvironment (TME), mainly in cancer and immune cells.^7,10–12^ Intratumoural microorganisms can significantly change the biology of different cell compartments, affecting the occurrence, development and metastasis of tumours and antitumour immunity.^7,13^

**Fig. 1:**
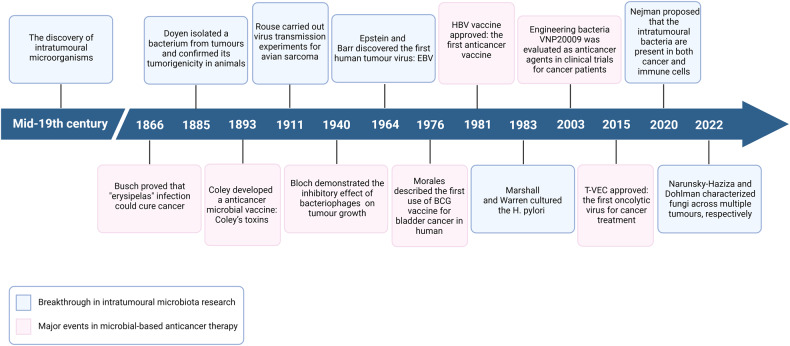
**Milestone events of intratumoural microbiota**. The key findings on intratumoural microbiota and the major achievements of microbial-based anticancer therapy were reviewed retrospectively. Created with BioRender.com

So far, many important discoveries about intratumoural microbiota have been reported (Fig. 1). As early as in the mid-19th century, microbiologists discovered the presence of a variety of microorganisms in tumours. In 1885, Doyen isolated a bacterium, *Micrococcus neoformans*, from different tumours and confirmed its tumorigenicity in animals.^14^ However, due to the limited sterile conditions available at that time, this findings were questioned. In the early 20th century, Rouse discovered that avian sarcoma can be transmitted in animals through filtered cell-free tumour extracts which suggested that causative agent in the tumour extracts, roussarcoma virus, could induce cancer, thereby establishing viruses as the causal agents of cancer for the first time.^15^ In 1964, Epstein and Barr discovered the first human virus particle in Burkitt’s lymphoma—Epstein-Barr virus (EBV).^16^ Since then, evidence that viral infections cause cancer in humans began to emerge. In 1983, Marshall and Warren cultured *H. pylori* and further established its role in gastric cancer aetiology.^17,18^ In 2020, the most rigorous and comprehensive survey of bacteria in seven human tumour samples by Nejman et al. revealed that different cancer types involve different bacterial species.^7^ Subsequently, Narunsky-Haziza and Dohlman separately characterised fungi in human cancer specimens from multiple tumour types to further explore the role of intratumoural fungi in cancer diagnosis and prognosis.^11,12^

Meanwhile, cancer treatment methods based on microbial intervention have also been developed. In 1866, Busch purposefully infected cancer patients with erysipelas and found that the tumours of the patients subsided.^19^ In 1893, Coley invented an anticancer drug microbial vaccine—Coley toxin— which has been observed to alleviate advanced cancers in some cases.^20^ As similar approaches became more widely researched, numerous anticancer vaccines emerged in the following decades.^21^ In addition, since Bloch demonstrated the interaction of bacteriophages with malignant cells and their ability to inhibit tumour growth in 1940,^22^ scientists have also started paying attention to the potential of these dynamic viral entities to treat cancer. In 1981, the hepatitis B virus (HBV) vaccine was approved as the first anticancer vaccine, saving millions of potential victims from developing hepatocellular carcinoma (HCC).^21^ By now, far beyond natural microbes, scientists have shifted their sights to modifying bacteria and viruses to fight cancer.

Based on the need for a comprehensive and in-depth understanding of the current research progress in intratumoural microbiota, this review summarises the characteristics and emerging functions of intratumoural microorganisms in various tumours as well as their effects on cancer development and antitumour immunity. We also discuss prospective applications of intratumoural microbiota in cancer prognosis and therapy. These findings complement the results of previous studies on intratumoural microorganisms and may provide potential theoretical support for future research on intratumoural microbiota-targeted cancer treatments.

## Characteristics of intratumoural microbiota

### Colonisation of the tumour by microorganisms

**Fig. 2:**
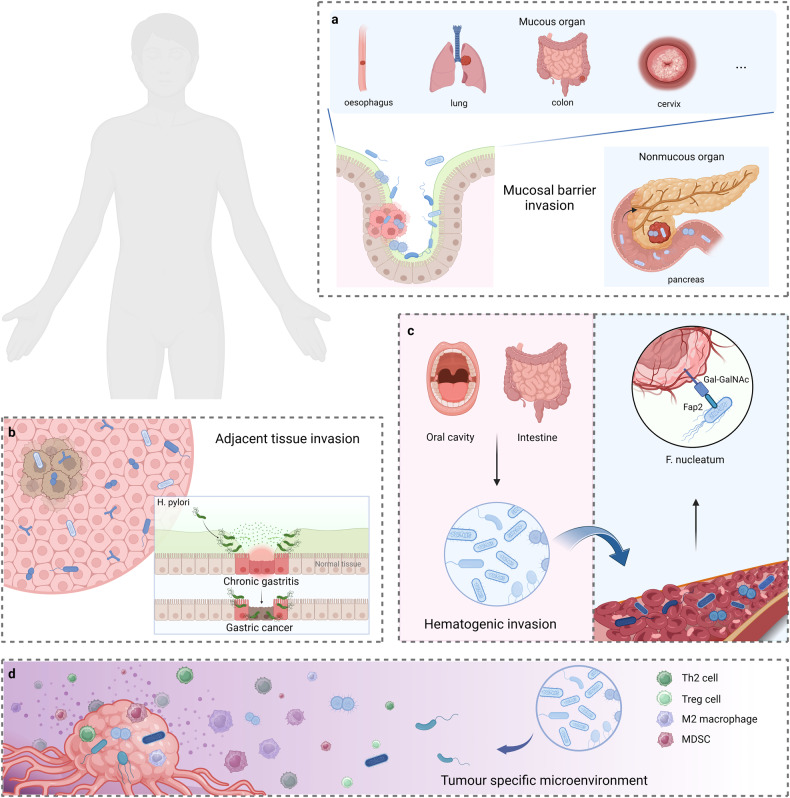
**The potential origins of intratumoural microbiota**. **a** Mucosal barrier invasion. Microorganisms may invade the tumour through the damaged mucosa. **b** Adjacent tissue invasion. the microbiome community between the tumour and adjacent normal tissue share many similarities. **c** Hematogenic invasion. microorganisms from the oral cavity, intestine, and other potential locations may be carried to the tumour locations and colonise the tumour through destroyed blood vessels. **d** Attraction of tumour specific microenvironment. immunosuppressive, hypoxic, and metabolic nutrient-enriched environments in tumours may enhance microbial colonisation. Created with BioRender.com

There are three possible origins of intratumoural microorganisms (Fig. 2). The first is mucosal barrier invasion, in which microorganisms colonising the mucosa may invade the tumour through the damaged mucosa.^23,24^ Tjalsma et al. proposed a bacterial driver–passenger model in which “driver” bacteria such as the genus *Bacteroides* and the family *Enterobacteriaceae* colonise the intestine and drive tumorigenesis. With microenvironment change, driving bacteria are gradually replaced by “passenger” bacteria, including opportunistic pathogens and commensal or probiotic bacteria, which further affect tumour progression.^25^ Many intratumoural microbiota have been found to colonise mucosal organs, such as the oesophagus, lung, colon, and cervix. Moreover, the intratumoural microbiota in non-mucosal organs, such as the pancreas, has also been found to translocate from the intestinal tract with impaired mucosal barriers and into the pancreas through the pancreatic duct, thereby reshaping the TME and increasing susceptibility to microbial translocation.^26^ The second is adjacent tissue invasion, where the microbiome communities between the tumour and adjacent normal tissue share many similarities.^7,27,28^ Moreover, many studies have found that viral infections^29^ and specific bacterially mediated chronic inflammation,^30^ such as *Helicobacter pylori* (*H. pylori*) and gastritis, can eventually evolve into tumours. Nonetheless, the origin of microorganisms in normal tissues of most organs remains unclear, and these microbes may also disseminate from the tumour site; therefore, further research is required to confirm this hypothesis. Finally, the third is hematogenic invasion, in which microorganisms from the oral cavity, intestine, and other potential locations may be carried to the tumour locations and colonise the tumour through destroyed blood vessels.^31^ Abed et al. reported that intravenously injected *Fusobacterium nucleatum* (*F. nucleatum*) interacts with the host polysaccharide D-galactose-β(1–3)-N-acetyl-D-galactosamine (Gal-GalNAc) in a lectin Fap2-dependent manner to localise to mouse tumour tissues, indicating that fusobacteria reached colon adenocarcinomas via a hematogenous pathway.^32^ A similar result was found in mouse mammary tumours.^33^ However, intestinal localisation of these tumours does not fully summarise the complex histological features of human colorectal adenomas. Furthermore, specific microenvironments in tumours may enhance microbial colonisation, such as immunosuppressive, hypoxic, and metabolic nutrient-enriched environments.^34^ However, these conjectures must be confirmed through holistic metagenomic sequencing and genetic identification.

### Diversity of intratumoural microbiota

The structure and abundance of the intratumoural microbial population vary substantially across different types, subtypes, and stages of cancer. Here, we have summarised the intratumoural microbiota in a few cancers to understand their role in cancer progression (Table 1).

**Table 1:** Characterization of the intratumoural microbiota in various cancers

Cancer types	Microbiome compositions	Quantitative dynamics	Function
**Lung cancer**	Genus *Modestobacter*^36^	Increase	**—**
	Genus *Propionibacterium*, genus *Enterobacteriaceae*^36^	Dcrease	**—**
	Genus *Blastomyces*^11^	Increase	**—**
	Class *Agaricomycetes*, genus *Aspergillus*^12^	Increase	**—**
	Genus *Acidovorax*^37^	Increase	Related to tumours with high TP53 mutation;
	Genus *Klebsiella*, genus *Anaerococcus*^37^	Increase	**—**
	Genus *Acinetobacter*, genus *Brevundimonas*, genus *Propionibacterium*^38^	Increase	**—**
	Phylum *Cyanobacteria*^39^	Increase	**—**
	Genus *Veillonella*, genus *Megasphaera*^40^	Increase	As the diagnostic biomarker of tumour;
	Family *Coriobacteriaceae*, genus *Pasteurella*^201^	**—**	Related to the number of CD8 + T cells and M2 macrophages;
	Species *Nontypeable Haemophilus influenzae*^188^	Increase	Released IL-17C and recruited the neutrophils;
**Liver cancer**	Hepatitis B virus^43,130,179,203^	Increase	Integrated viral genome into the host chromosome; induced m6A modification of RNA Recruited Treg cells;
	Hepatitis C virus^43,204^	Increase	Recruited Treg cells;
	Species *Helicobacter pylori*^48–50^	Increase	**—**
	Order *Gammaproteobacteria*^53^	Increase	**—**
	Family *Streptococcaceae*, genus Lactococcus^53^	Increase	As the hallmark groups of cirrhosis hepatocellular carcinoma;
	Family *Enterobacteriaceae*^54^	Increase	**—**
	Family *Caulobacteraceae*, family *Rickettsiaceae*^54^	Decrease	**—**
	Species *Paraburkholderia fungorum*^55^	Decrease	Related to antitumor activity;
**Colorectal cancer**	Species Enterotoxigenic *Bacteroides fragilis*^56,140,186^	Increase	Secreted carcinogenic toxins; initiated pro-inflammatory signalling cascade;
	Genus *Fusobacterium*^57–59,182^	Increase	Promoted the polarization of M2-like macrophages;
	Genus *Lactococcus*, genus *Bacteroides*, genus *Prevotella*, genus *Streptococcus*^59^	Increase	**—**
	Genus *Pseudomonas*, genus *Escherichia-Shigella*^59^	Decrease	**—**
	Species *Fusobacterium nucleatum*^60,141,166–168,174,190,199,210,211,242,248^	Increase	Related to advanced-stage tumour; induced histone modification; upregulated DNA methyltransferases; inhibited autophagic process; activated β-catenin signalling; aggregated tumour-infiltrating myeloid cells; activated TIGIT and CEACAM1 receptors expressed on immune cells; induced EMT; upregulated ICAM1 and promoted the adhesion of cancer cells to endothelial cells;
	Genus *Bifidobacterium*, genus *Romboutsia*^62^	Increase	Related to left-sided colon cancers;
	Genus *Haemophilus*, genus *Veillonella*^62^	Increase	Related to right-sided colon cancers;
	Phylum *Ascomycota* Class *Malasseziomycetes*^65^	Increase	**—**
	Class *Saccharomycetes*, class *Pneumocystidomycetes*^65^	Dcrease	**—**
	Species *Escherichia coli*^140,249^	Increase	Secreted carcinogenic toxins; related to metastasis;
	Species Enteropathogenic *Escherichia coli*^144^	Increase	Disrupted mechanisms of DNA mismatch repair;
	Species *Hungatella hathewayi*^168^	**—**	Upregulated DNA methyltransferases;
	Genus *Akkermansia*^194^	**—**	Increased IL-17 production and B cell infiltration;
	Genus *Candida*^11^	Increase	Involved in the downregulation of genes mediating cellular adhesion;
**Gastric cancer**	Species *Helicobacter pylori*^68,69,76,145,161,162,212–214^	Increase	Related to early-stage of tumours; disrupted DNA mismatch repair mechanisms; inducted abnormal DNA methylation; activated CEACAM1 on immune cells; upregulated PD-L1 expression;
	Genus *Prevotella*, genus *Streptococcus*, genus *Veillonella*, genus *Haemophilus* genus *Neisseria*^73^	Increase	**—**
	Genus *Helicobacter*^73,227^	Decrease	Metabolic regulation;
	Genus *Lactobacillus*^74,227^	Increase	Related to tumour progression; metabolic regulation;
	Phylum *Nitrospirae*, family *Lachnospiraceae*, genus *Escherichia-Shigella*, species *Burkholderia fungorum*^74^	Increase	Related to tumour progression;
	Genus *Oceanobacter*, genus *Methylobacterium*, genus *Syntrophomonas*^75^	Increase	**—**
	Species *Propionibacterium acnes*, species *Prevotella copri*^76^	Increase	Related to early-stage of tumours;
	Phylum *TM7*, genus *Porphyromonas*, genus *Neisseria* species *Streptococcus sinensis*^77^	Decrease	Related to benign gastric disease;
	Family *Lachnospiraceae*, species *Lactobacillus coleohominis*^77^	Increase	Related to malignant gastric disease;
	Epstein-Barr virus^70–72^	Increase	Related to DNA hypermethylation;
	Order *Clostridium*, family *Comamonadaceae*, genus *Moryella*, genus *Vibro*, genus *Paludibacter*, genus *Agrobacterium*^30^	Increase	Monitor the risk of gastric cancer development;
	Species *Kytococcus sedentarius*, species *Actinomyces oris*^163^	Increase	Inducted abnormal DNA methylation;
**Breast cancer**	Genus *Pseudomonas*, genus *Proteus*^81^	Increase	**—**
	Species *Methylobacterium radiotolerans*^82,83^	Increase	**—**
	Species *Sphingomonas yanoikuyae*^82^	Decrease	**—**
	Genus *Methylobacterium*^84^	Decrease	**—**
	Genus *Cladosporium*^12^	Increase	**—**
	Genus *Lactobacillus*^85,225^	Increase	Metabolic regulation; enhanced the resistance of tumour cells to flow shear stress;
	Genus *Streptococcus*, genus *Staphylococcus*^85^	Increase	Enhanced the resistance of tumour cells to flow shear stress;
	Family *Streptococcaceae*^88^	Increase	**—**
	Genus *Bosea*^88^	Increase	Related to tumour progression;
	Species *Escherichia coli*, species *Staphylococcus epidermis*^143^	Increase	Caused double-stranded DNA breaks;
	Genus *Fusobacterium*, genus *Atopobium*, genus *Hydrogenophaga*, genus *Gluconacetobacter*^225^	Increase	Metabolic regulation;
	Species Enterotoxigenic *Bacteroides fragilis*^246^	Increase	Enhanced stemness potential and metastatic progression;
	Species *Fusobacterium nucleatum*^33,252^	Increase	Related to metastasis;
**Pancreatic cancer**	Phylum *Proteobacteria*^91^	Increase	**—**
	Species *Helicobacter pylori*^92,93^	Increase	Related to activation of molecular pathways for tumour growth and progression;
	Genus *Pseudomonas*^91,94^	Increase	Related to carcinogenesis;
	Genus *Elizabethkingia*^91^	Increase	**—**
	Genus *Acinetobacter*, genus *Sphingopyxis*^94^	Increase	Related to carcinogenesis;
	Genus *Malassezia*^95^	Increase	**—**
**Oral cancer**	Genus *Fusobacterium*^101,112^	Increase	**—**
	Species *Fusobacterium nucleatum*^142,229^	Increase	Caused double-stranded DNA breaks; Promoted GLUT1 upregulation and lactic acid accumulation;
	Species *Porphyromonas gingivalis*^102–105,154,155,244^	Increase	Related to the formation of DNA adducts or the inhibition of DNA repair enzymes; Related to the expression of EMT-related transcription factors;
	Genus *Prevotella*^106^	Icrease	**—**
	Species *Prevotella intermedia*^107^	Increase	Related to carcinogenesis;
	Species *Treponema denticola*^108,109^	Increase	**—**
	Species *Streptococcus anginosus*^110,111,155^	Increase	Related to the formation of DNA adducts or the inhibition of DNA repair enzymes;
	Genus *Streptococcus*^112^	Decrease	**—**
	Species *Pseudomonas aeruginosa*, *Campylobacter sp*. Oral taxon 44^113^	Increase	**—**
	Human papillomavirus^98^	Increase	**—**
	Epstein-Barr virus^99^	Increase	**—**
	Herpes Simplex Virus Type 1^100^	Increase	**—**
	Species *Candida albicans*, species *Candida etchellsii*, species *Hannaella luteola-like*^114^	Increase	**—**
**Head-and-neck squamous cell carcinomas**	Genus *Parvimonas*^116^	Increase	**—**
	Genus *Actinomyces*^116^	Decrease	**—**
	Human papillomavirus types 16^117,134^	Increase	Inhibited the cGas-STING pathway;
**Oesophageal cancer**	Species *Campylobacter conisus*^189^	Increase	Upregulated PRRs and aggregated IFI16 inflammasome;
**Nasopharyngeal carcinoma**	Genus *Corynebacterium*, genus *Staphylococcus*^28^	Increase	**—**
	Epstein-Barr virus^119,217^	Increase	Downregulated IDO expression;
**Ovarian carcinoma**	Phylum *Aquificae*, phylum *Planctomycetes*^120^	Increase	**—**
	Phylum *Crenarchaeota*^120^	Decrease	**—**
	Human papillomavirus types 16, 18, and 45^121^		Related to advanced-stage tumours;
**Endometrial cancer**	Genus *Bacteroides*, genus *Faecalibacterium*^122^	Increase	**—**
	Genus *Staphylococcus*, genus *Blautia*, genus *Parabacteroides*^122^	Decrease	**—**
**Cervical cancer**	Genus *Gardnerella*, genus *Prevotella*, genus *Streptococcus*, genus *Atopobium*^226^	Increase	Metabolic regulation;
	Human papillomavirus^131,205,208,209^	Increase	Integrated viral genome into the host chromosome; recruited Treg cells; inhibited cytotoxic T and NK cell activation; upregulated PD-L1 expression;
**Prostatic cancer**	Species *Cutibacterium acnes*^123,124^	Increase	**—**
	Species *Staphylococcus aureus*^202^	Increase	Recruited Treg cells;
**Bladder cancer**	Species *Escherichia coli*, species *Butyrate-producing bacteria SM4/1*, a species of *Oscillatoria*^243^	Increase	Related to the expression of EMT-related genes;
**Pituitary neuroendocrine tumour**	Order *Clostridiales*, family *Fusobacteriaceae*, family *Tissierellaceae*, family *Aerococcaceae*, family *Corynebacteriaceae*, family *S24-7*, *F16*^125^	Increase	Related to different clinical phenotypes of tumour;
**Burkitt’s lymphoma**	Epstein-Barr virus^126^	Increase	**—**
**Hodgkin’s lymphoma**	Epstein-Barr virus^126^	Increase	**—**
**NK cell and T cell lymphomas**	Epstein-Barr virus^126^	Increase	**—**
	Human T-lymphotropic virus type 1^139^	Increase	Inhibited DNA repair pathway.
**Lymphocytic leukaemia**	Human endogenous retroviruses^118^	Increase	**—**

#### Lung cancer

As the mucosa is in primary contact with the external environment, the lungs are exposed to microbes and environmental factors and harbour diverse microbes.^35^ In lung cancer tissues, the prevalence of *Modestobacter* was higher than that in adjacent normal tissues, while the prevalence of *Propionibacterium* and *Enterobacteriaceae* was lower.^36^ Dohlman et al. found that abundant *Blastomyces* existed in lung tumours.^11^ In addition, current smokers had higher intratumoural fungal diversity and abundance of *Aspergillus* and *Agaricomycetes* than never smokers.^12^ The intratumoural microbiota is also related to the histological subtype and tumour stage of lung cancer. Compared to lung adenocarcinoma, lung squamous cell carcinoma microbiota is more diverse. The relative abundances of *Acidovorax*, *Klebsiella*, and *Anaerococcus* in squamous cell carcinoma were elevated, especially in patients with tumour protein p53 (TP53) mutations, where *Acidovorax* was more abundant.^37^ In adenocarcinomas*, Acinetobacter*, *Brevundimonas*, and *Propionibacterium* were more abundant.^38^ Furthermore, Apopa et al. discovered that the phylum *Cyanobacteria* was more enriched in adenocarcinoma.^39^ Additionally, it has been found that the genera *Veillonella* and *Megasphaera* have a high area under the curve for predicting lung cancer.^40^

A few studies have aimed to describe the intratumoural micropopulation associated with lung cancer. Most of the samples used in these studies were based on indirect specimens of bronchoalveolar lavage fluid (BALF), sputum, and airway brushing tissue, which could be problematic because the upper and lower respiratory tracts have distinct microbial populations that might result in cross-contamination.^41^ Therefore, surgical specimens would provide a more precise evaluation of microorganisms in lung cancer.

#### Liver cancer

Primary liver cancer (PLC) is a malignancy that originates from malignant hepatocellular tumours and precursors such as HCC, the main type of PLC.^42^ PLC generally progresses from chronic liver diseases, such as viral hepatitis, as ~56% of liver cancers are associated with HBV and 20% with the hepatitis C virus (HCV).^43^ The interaction between microbiota and PLC has been intensively investigated because of the gut-liver axis.^44–47^ However, studying microorganisms in liver tumours remains limited. *H. pylori* and similar species have been identified in patients with HCC liver tissues.^48–50^ Although in vitro studies have suggested the potential connection between *H. pylori* and liver cancer development, no evidence has been obtained showing that this species directly contributes to tumorigenesis.^51,52^ Huang et al. found microbes present in hepatocytes and erythrocytes, and the abundance of *Gammaproteobacteria* in cancerous tissues was considerably higher than that in normal tissues. In particular, compared with that in non-cirrhosis HCC, *Streptococcaceae* and *Lactococcus* were significantly increased in cirrhosis HCC, suggesting that they could be used as marker taxa for cirrhosis HCC.^53^ Qu et al. demonstrated that the abundance of *Enterobacteriaceae* was substantially higher in the HCC group, whereas the abundances of *Caulobacteraceae* and *Rickettsiaceae* were substantially lower in the combined hepatocellular carcinoma and intrahepatic cholangiocarcinoma (cHCC‐ICC) group.^54^ Moreover, the abundance of *Paraburkholderia fungorum* in ICC was higher in paraneocancerous tissues than in cancerous tissues, and the value was inversely correlated with carbohydrate antigen 199 (CA199) levels. The results of in vitro and in vivo experiments further indicated that the fungus had antitumour activity.^55^ Currently, studies on the intratumoural microorganisms of HCC are limited, and further investigation is needed to identify the significance of other microbiota in HCC.

#### Colorectal cancer

*Fusobacterium* and enterotoxigenic *Bacteroides fragilis* (ETBF) have been demonstrated to be carcinogenic factors in the advancement of colorectal cancer (CRC).^56–58^ Unlike normal tissues, *Lactococcus*, *Bacteroides*, *Fusobacterium*, *Prevotella*, and *Streptococcus* were more abundant in tumour tissues. *Pseudomonas* and *Escherichia-Shigella* were substantially enriched in adjacent normal tissues compared to those in tumour tissues.^59^ Yamamoto et al. also discovered a varied abundance of *F. nucleatum* in the progression of CRC, with the highest prevalence in stage III/IV tumours.^60^ However, another study reported that *F. nucleatum* was not detected in most CRC samples and that there were no substantial differences in the microbiota between tumours and adjacent tissues.^61^ This may be due to differences in patient selection and techniques used among the studies. In addition, distinctions between left and right colon cancers were detected, where the loadings of *Bifidobacterium* and *Romboutsia* were higher in left-sided colon cancers (LSCCs), whereas *Haemophilus* and *Veillonella* were higher in right-sided colon cancers (RSCCs).^62^ This may partially explain the difference in the biological subtypes of CRC between the LSCCs and RSCCs, with increased levels of the “microsatellite unstable/immune” consensus molecular subtype (CMS)1 and the “metabolic” CMS3 identified in RSCCs.^63^ Regarding fungi, Ascomycota, Glomeromycota, and Basidiomycota were identified as the predominant phyla between adenomas and adjacent tissues,^64^ and the abundance of Ascomycota was higher in patients with CRC, with an increase in *Malasseziomycetes* and a decrease in *Saccharomycetes* and *Pneumocystidomycetes*.^65^

Several studies on the CRC microbiome utilised faecal samples due to the simple and non-invasive sample collection procedure. However, intestinal mucosal tissue samples are more suitable for assessing the physiopathology of CRC, and distinct microbiome patterns in mucosal and faecal samples have also been reported.^66,67^ The composition of CRC-related micropopulations has not yet been unified and requires further research.

#### Gastric cancer

*H. pylori* is the most predominant microorganism detected in gastric cancer (GC) and has been implicated in promoting premalignant lesions that can ultimately progress to GC.^68,69^ EBV was found within malignant epithelial cells in 9% of GC.^70^ EBV-GC may be the most common type of EBV-associated cancer, highly correlated with cyclin-dependent kinase inhibitor 2 A (*CDKN2A*) promoter hypermethylation.^71,72^ Shao et al. found that the microbial diversity in GC cells was considerably greater than that in benign stomach lesions. Specifically, the genera *Prevotella*, *Streptococcus*, *Veillonella*, *Haemophilus*, and *Neisseria* were more abundant, whereas *Helicobacter* was less abundant.^73^ Moreover, GC tissues have a high load of potentially carcinoma-promoting bacteria, including *Lactobacillus*, *Escherichia-Shigella*, *Lachnospiraceae*, *Nitrospirae*, and *Burkholderia fungorum*.^74^ Peng et al. observed that the *Oceanobacter, Methylobacterium*, and *Syntrophomonas* genera were enriched in tumour tissue, and the intratumoural *Methylobacterium* was considerably correlated with a poor prognosis in GC patients.^75^ Recent research has demonstrated that as GC progresses, the abundance of *H. pylori* may gradually reduce, and the microbiota diversity may also change. In the early-stages of GC, the numbers of *H. pylori*, *Propionibacterium acnes*, and *Prevotella copri* were higher than that in non-cancer patients.^76^ In addition, the microbiome of patients at different histological stages, from gastritis to precancerous lesions to stomach cancer, also showed changes. The abundance of *TM7*, *Porphyromonas*, *Neisseria*, and *Streptococcus sinensis* decreased with disease progression, while that of *Lactobacillus coleohominis* and *Lachnospiraceae* were reversed.^77^ In addition, a longitudinal prospective study identified a total of six microbial taxonomic features, namely the *Moryella* genus, *Vibro* genus, *Comamonadaceae* family, *Paludibacter* genus, *Agrobacterium* genus, and *Clostridium* order, at baseline that could be used to indicate the risk of future GC development.^30^

#### Breast cancer

Breast cancer (BC) is the most widespread malignancy among women.^78^ The relationship between the microbiota and carcinogenesis has been evaluated to determine its function in the initiation and progression of BC.^79,80^ Breast tumours have the greatest bacterial diversity and abundance among all tumours.^7^ Tzeng et al. discovered that the genera *Pseudomonas* and *Proteus* were highly enriched in BC tissues.^81^ Xuan et al. demonstrated that *Methylobacterium radiotolerans* was increased in tumour tissue, while *Sphingomonas yanoikuyae* was increased in normal tissue. Moreover, the tumour stage was inversely associated with total bacterial load at the tumour site, which may provide clues for BC diagnosis.^82^ Another study also revealed an increased abundance of *Methylobacterium radiotolerans* in the tumour sentinel lymph nodes.^83^ However, Wang et al. demonstrated a decreased abundance of *Methylobacterium* in BC tissues.^84^ Narunsky-Haziza et al. identified that the *Cladosporium* genus was increased in the BC of patients ≥50 years old.^12^ The unique intratumoural microbiota present in BC tissues, mainly comprising *Lactobacillus*, *Streptococcus*, and *Staphylococcus*, is a potential factor contributing to tumour metastasis.^85^ Multiple investigations have found that the microbial community varies according to subtype.^86,87^ The family *Streptococcaceae* is more abundant in the triple-negative BC subtype, and the abundance of the genus *Bosea* increases with the progression of the tumour.^88^

#### Pancreatic cancer

Pancreatic cancer (PC) is a malignant cancer characterised by a dismal prognosis; pancreatic ductal adenocarcinoma (PDAC) accounts for most of PC.^89^ Recent research has revealed the existence of bacteria in the pancreas; PC tissues contain a larger proportion of bacteria than normal pancreatic tissues.^90^ *Proteobacteria* are the most prevalent intratumoural microorganisms in PDAC, similar to the normal duodenal microbial composition.^91^ Studies have suggested that *H. pylori* colonised PC cells and was associated with the activation of molecular pathways for tumour initiation and development.^92,93^ However, the subspecies of *Helicobacter* found in pancreatic and gastroduodenal tissues were different, suggesting that *Helicobacter* in the pancreas may not have migrated from the gastroduodenum.^92^ Moreover, the microbes in PDAC are completely distinct from those in normal pancreatic tissues, especially regarding *Pseudomonas* and *Elizabethkingia*, which were highly abundant in tumours.^91^ Basal-like tumours, a highly aggressive PDAC subtype, were found to have distinctive intratumoural microbiota, and increased abundance of *Acinetobacter*, *Pseudomonas*, and *Sphingopyxis* was strongly associated with carcinogenesis.^94^ In addition, the fungal population of PDAC tumours was found to be significantly enriched with *Malassezia*.^95^

#### Oral cancer

The oral microbiome hosts >750 common oral species.^96^ The normal oral microbiome mainly comprises aerobes; the percentage of anaerobes increases with the development of oral cancer (OC). Oral squamous cell carcinoma (OSCC) constitutes 90% of epithelial malignancies in the oral cavity.^97^ Human papillomavirus (HPV) has been recognised as a potential contributor to OSCC, with HPV type 16 being the most significant subtype. Approximately 25–35% of OSCCs are attributed to HPV infection.^98^ Other oncogenic viruses, such as EBV and Herpes Simplex Virus Type 1 (HSV-1), have also been demonstrated to be associated with OSCC.^99,100^ *F. nucleatum* is the natural flora present in oral mucosa that has been implicated in the development of oral malignancies. Nagy et al. observed a higher abundance of *Fusobacterium* in OSCC tissue than in normal mucosal tissue.^101^ *Porphyromonas gingivalis* (*P. gingivalis*) is another independent and critical risk factor for OC.^102,103^ Katz et al. detected a significant enrichment of *P. gingivalis* in gingival squamous cell carcinoma.^104^ Similarly, Chang et al. revealed that the level of *P. gingivalis* in OSCC tissue was higher than that in normal tissue; they also found a positive correlation between *P. gingivalis* infection and advanced-stage, poor differentiation, and lymph node metastasis among OSCC patients.^105^ Another study revealed that *Prevotella* were enriched in OSCC tissue.^106^ In particular, Zhang et al. found that the abundance of *Prevotella intermedia* was significantly increased in OSCC; functional prediction further suggested that the bacteria were associated with carcinogenesis.^107^ Moreover, studies have shown that *Treponema denticola* is closely correlated with OSCC and oropharyngeal squamous cell carcinoma (OPSCC).^108,109^ The aerobic bacterium *Streptococcus* has also been found to be associated with OC. Sasaki et al. showed that the levels of *Streptococcus anginosus* (*S. anginosus*) were elevated in patients with OC.^110^ Rai et al. reported similar results.^111^ Some studies, however, have reported contrasting results. Su et al. found that *Fusobacterium* was substantially enriched at tumour sites, while *Streptococcus* showed the opposite results.^112^ Besides, *Pseudomonas aeruginosa* and *Campylobacter sp*. Oral taxon 44 were also abundant in OSCC.^113^ Moreover, Perera et al. found that *Candida albicans*, *Candida etchellsii*, and a *Hannaella luteola-like* species were relatively abundant in OSCC.^114^

Additionally, the oral microbiota changed with the progress of OC. Yang et al. demonstrated that as cancer progressed, the prevalence of *Fusobacterium* increased, while that of *Streptococcus*, *Haemophilus*, *Porphyromonas*, and *Actinomyces* decreased.^115^

#### Other cancers

In addition to the cancers reported above, multiple studies have also shown the presence of microbiota in other tumours. In an investigation of head-and-neck squamous cell carcinomas (HNSCCs), *Actinomyces* was significantly reduced, while *Parvimonas* was elevated relative to that in normal tissues.^116^ Another study demonstrated that HPV 16 was detected in HNSCCs,^117^ and a significant exclusivity of HPV and driver mutations in *TP53*, *CDKN2A*, and *telomerase reverse transcriptase* was exhibited in the tumour.^118^ In addition to EBV,^119^ a recent study found that some bacteria, mainly *Corynebacterium* and *Staphylococcus*, existed in nasopharyngeal carcinoma (NPC) tumour tissues, and the total intratumoural bacterial load was negatively correlated with prognosis.^28^ Among other reproductive system tumours, the microbiota in ovarian cancer tissue consisted of increased *Aquificae* and *Planctomycetes* abundance and decreased *Crenarchaeota* abundance.^120^ Compared to normal adjacent tissues, ovarian carcinoma contained a higher proportion of HPVs, and high-risk HPV types 16, 18, and 45 were significantly correlated with advanced-stage tumours.^121^ *Bacteroides* and *Faecalibacterium* were particularly associated with endometrial cancer, while *Staphylococcus*, *Blautia*, and *Parabacteroides* were more associated with benign uterine disease.^122^ Certain bacterial species, especially *Cutibacterium acnes*, can persist in prostatic tissue specimens.^123,124^ Intracranial tumours, such as glioblastomas^7^ and pituitary neuroendocrine tumours (PitNETs),^125^ were found to contain intratumoural microorganisms, and the abundance of microorganisms in different subtypes of the PitNETs was also different, which *Fusobacteriaceae*, *Tissierellaceae*, and *Aerococcaceae* were substantially enriched in adrenocorticotropic hormone-secreting PitNET (ACTH-PitNET) tissues and *Corynebacteriaceae*, *S24-7*, *Aerococcaceae*, *Clostridiales*, and *F16* were more enriched in growth hormone-secreting PitNET (GH-PitNET) tissues. Moreover, EBV was present in the blood system’s tumour cells, such as Burkitt’s lymphoma, Hodgkin’s lymphoma, and some natural killer (NK) and T cell lymphomas.^126^ Human endogenous retroviruses (HERVs) have been found in chronic lymphocytic leukaemia, where ERV1 was strongly expressed.^118^ However, the relationships between intratumoural microbiota and other types of cancers have not been thoroughly studied, and further research is required.

## Role of intratumoural microbiota in the development of cancer

**Fig. 3:**
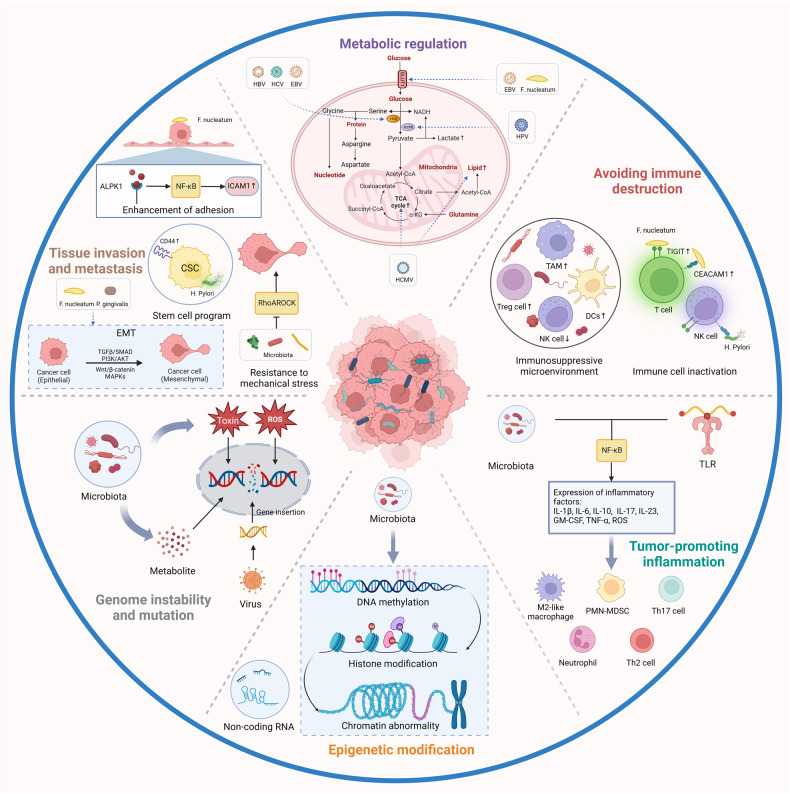
**The role of intratumoural microbiota in the development of cancer**. The potential effects of the microbiome on the cancer remain elusive. Six major mechanisms have been proposed to explain how the intratumoural microbiota influence the initiation and advancement of cancer, including genome instability and mutation, epigenetic modification, chronic inflammation, immune evasion, metabolic regulation, activation of invasion and metastasis. TAM, tumour-associated macrophage; HK2, hexokinase 2; TCA, tricarboxylic acid. Created with BioRender.com

Cancer cells influence disease progression by maintaining proliferation, evading growth inhibition, resisting cell death, enabling replicative immortality, inducing angiogenesis, and activating invasion and metastasis.^127,128^ Although the potential role of the microbiome in the initiation and advancement of cancer remains elusive, it could be associated with modulating the most relevant tumour-promoting functions between malignant and non-malignant cells. Understanding these mechanisms is crucial for cancer prediction and treatment (Fig. 3).

### Genome instability and mutation

The induction of genomic instability and mutation is one of the carcinogenic mechanisms of the microbiome. More than 10% of human malignancies are primarily caused by oncoviruses.^129^ Various studies have suggested that oncoviruses cause cancer by integrating the viral genome into the host chromosome and triggering genetic mutations, such as HPVs in cervical, head-and-neck, and several other cancers, and HBV in liver cancer.^130–132^ Integrated deoxyribonucleic acid (DNA) also leads to viral oncoprotein production, which modulates host signalling pathways and alters genes’ and ribonucleic acid (RNA) expression. In mouse models, the HPV E7 oncoprotein directly inhibits the cyclic guanosine monophosphate (MP)-adenosine MP synthase (cGas)-stimulator of interferon genes (STING) pathway and specifically reduces the expression of genes encoding type I interferon and pro-inflammatory factors, thereby driving immune escape in multiple HPV-related tumours.^133,134^ Moreover, EBV and Kaposi sarcoma-associated herpesvirus (KSHV) oncoproteins can upregulate oncogenic cellular proteins and microRNAs in mouse, downregulate tumour suppressors, and trigger signalling pathways, such as the nuclear factor kappa-light-chain-enhancer of activated B cells (NF-κB) pathway, to drive the proliferation and transformation of B cells and endothelial cells.^135–138^ Human T-lymphotropic virus type 1 (HTLV-1), the retrovirus that triggers adult T cell leukaemia, inhibits the DNA repair pathway through the HTLV-1 Tax protein, leading to genome instability and the accumulation of carcinogenic mutations.^139^

Certain carcinogenic bacteria, such as pks+ *Escherichia coli* (*E. coli*) and ETBF, encode and secrete carcinogenic toxins that induce DNA damage, which results in faster tumour onset and greater mortality.^140^ Moreover, FadA, a key adhesin secreted by *F. nucleatum*, promotes E-cadherin/β-catenin activation to upregulate checkpoint kinase 2 (CHK2), causing DNA damage in mouse CRC cells.^141^ *F. nucleatum* infection promotes OSCC by causing Ku70/p53 pathway-dependent DNA double-strand breaks (DSBs).^142^ *E. coli* and *Staphylococcus epidermis* isolated from breast tumour tissue cause DSBs in HeLa cells.^143^ In addition, EspF-expressing enteropathogenic *E. coli* and *H. pylori* may interfere with DNA mismatch repair mechanisms, thereby aggravating genome instability and promoting tumorigenesis.^144,145^ However, further studies are required to identify the direct involvement of bacterial virulence factors (VirFs) in cancer induction. The fundamental role of these VirFs could be to induce DNA damage response and subsequently activate the immune system, which results in pro-inflammatory outcomes and forms a microenvironment conducive to cancer.^146^ Microbial activities can also elicit the generation of reactive oxygen species (ROS), hydrogen sulphide, and superoxide dismutase.^147–149^ ETBF toxin increases the expression of spermine oxidase (SMO) in HT29/c1 and T84 colonic epithelial cells, triggering the SMO-dependent production of ROS and activation of γ-H2A, which causes DNA damage.^56^

In addition, many microbial metabolites affect tumour development by promoting DNA damage. BC tissues harbour increased levels of β-glucuronidase, which has been identified as a carcinogenic enzyme.^150,151^ β-glucuronidase may release reactive intermediates from 2-amino-3-methylimidazo[4,5-f]quinoline to cause DNA damage in rats.^152^ *S. anginosus* and *P. gingivalis* can convert ethanol into acetaldehyde, resulting in the formation of DNA adducts or the inhibition of DNA repair enzymes, which might cause DNA damage and oral carcinogenesis.^153–155^

### Epigenetic modification

Epigenetic pathways also play an essential part in oncogenesis by aberrantly silencing tumour suppressor genes (TSGs) and activating oncogenes.^156^ According to reports, bacterial infections survive, replicate, and evade destruction by the host immune system by regulating the host epigenome.^157–160^ Induction of abnormal DNA methylation is the main pathway for *H. pylori* infection to induce gastric adenocarcinoma.^161,162^ Other gastric microbiota, such as *Kytococcus sedentarius* and *Actinomyces oris*, are also involved in this mechanism, which promotes the occurrence of gastric adenocarcinoma and metastasis and affects its prognosis.^163^ A study reported that the significantly rich microorganisms in the high-cell subtype thyroid cancer patients were associated with higher tumour suppressor gene methylation.^164^ A recent study compared intratumoural microbiome and DNA methylation profiles of HCC tissue and normal liver tissue, and correlation analysis showed that 10 metabolome-related microbiome groups were closely related to 25 methylation-related differentially expressed genes.^165^ The abundance of intratumoural *F. nucleatum* was linked to increased infiltration of macrophages and promoter CpG island hypermethylation of *CDKN2A* in CRC patients.^166^ However, the molecular mechanism underlying the host epigenetic changes induced by intratumoural microbiota has not yet been fully characterised. On the one hand, some studies have reported that microorganisms directly regulate host epigenetic modifications. Liu et al. reported that in vivo and in vitro *H. pylori* infection promoted guanine nucleotide-binding protein subunits β-44 (GNB4) demethylation by activating NF-κB to upregulate TET1, inducing the carcinogenic pathway.^161^ Besides, *F. nucleatum* upregulates the transcription of long non-coding RNA (lncRNA) enolase1-intronic transcript 1 (ENO1-IT1) through transcription factor SP1. Elevated ENO1-IT instructs KAT7 histone acetyltransferase to change the histone decorator pattern on its target genes, enhancing CRC glycolysis and tumorigenesis.^167^ *F. nucleatum* and *Hungatella hathewayi* could mediate *TSG* promoter hypermethylation by upregulating DNA methyltransferases in CRC.^168^ On the other hand, it was reported that microorganisms synthesised and metabolised abundant compounds that serve as epigenetic substrates and cofactors or regulate epigenetic enzymes, indirectly affecting host epigenetic modifications. For example, folate and other B vitamins (B2, B12) are the primary substrates for DNA and histone methylation.^169^ Microorganism-derived short-chain fatty acids (SCFAs) trigger genomic epigenetic changes by affecting the activities of histone acetylase and histone deacetylase.^170,171^ A recent study found that specific bacteria produce methionine in lung cancer patients,^172^ and they behave as the main methyl donors for nucleic acid and protein methylation, making epigenetic reprogramming of host cells possible.^173^ Moreover, microorganisms may activate other pathways that indirectly alter the epigenetics of host cells. Koi et al. found that chronic *F. nucleatum* infection produces ROS, which causes DNA damage and triggers MSH2/MSH6-dependent repair, resulting in DNA hypermethylation.^174^ Previous reviews have described the changes in various non-coding RNAs induced by bacterial infection and their role in modifying chromatin structure.^175–177^ However, few studies have focused on intracellular microorganisms in this area. Epigenetic regulation crosstalk between the virus and the host also occurs. Pietropaolo et al. reviewed the research progress on seven viruses causing cancer through epigenetic changes in host cells.^178^ Another review summarised several mechanisms by which HBV induces hepatocarcinogenesis by inducing m6A modification of RNA, including virus replication, immune escape, and carcinogenesis, indicating a complex interaction between the microbiome and host.^179^

In conclusion, multiple studies have demonstrated that intratumoural microorganisms (mainly viruses and bacteria) could directly or indirectly regulate host epigenetic modifications, including DNA modification, histone modification, RNA modification, and non-coding RNA. However, the molecular mechanisms underlying the host epigenetic changes induced by intratumoural microorganisms must be further explored.

### Chronic inflammation

Persistent chronic inflammation is correlated with the advancement of most cancer types.^180^ The intratumoural microbiome can activate inflammatory signalling pathways and cascades by interacting with pattern recognition receptors (PRRs), such as Toll-like receptors (TLRs), in the TME.

TLRs are expressed on a variety of immune cells, including macrophages, dendritic cells, B cells, certain types of T cells, as well as non-immune cells like fibroblasts and epithelial cells.^181^ *Fusobacterium* recognised by TLR4 enhanced the interleukin (IL)-6/phospho-signal transducer and activator of transcription 3 (p-STAT3)/c-MYC signalling pathway, resulting in M2-like macrophage polarisation and mouse CRC progression.^182^ Pushalkar et al. showed that the PDAC microbiota inhibits the type 1 T-helper (Th1) polarisation of cluster of differentiation (CD)4 + T cells and M1-like macrophage differentiation by activating TLR, thus generating a tolerant immune programme.^91^ Moreover, in a mouse colitis-associated tumorigenic model, Yang et al. found that microbial-derived lipopolysaccharide /TLR4 mediates the chemokine-dependent recruitment of monocyte-like macrophages to promote IL-1β production, further promoting Th17 cell expansion. This process may enhance intestinal permeability and allow the excessive release of microbial products, thus promoting M2-like macrophage differentiation, generating a positive feedback loop to attract immunosuppressive cells and forming a tolerogenic microenvironment.^183,184^ Another mouse model showed that microbial products elicited by tumorous epithelial barrier disruption activate tumour-infiltrating inflammatory dendritic cells (DCs), thereby inducing γδT17 cells polarisation, which can release a lot of IL-17, IL-8, granulocyte-macrophage colony-stimulating factor, tumour necrosis factor alpha (TNF-α), and other cytokines to promote inflammation. These cytokines further attract the accumulation of polymorphonuclear myeloid-derived suppressor cells (PMN-MDSCs), thus transforming the inflammatory microenvironment into an immunosuppressor microenvironment and promoting the progression of CRC.^185^ Moreover, ETBF toxin triggers a STAT3–NF-κB-dependent pro-inflammatory signalling cascade to release cytokines like IL-17 and IL-23, which has been demonstrated to attract pro-tumoural myeloid cells and promote distal colon tumorigenesis.^186^ Park et al. demonstrated that *F. nucleatum* and *Aggregatibacter actinomycetemcomitans* activate TLR2 and TLR4 and downstream NF-κB in bone marrow-derived macrophages to stimulate IL-6 production in mouse.^187^ *Nontypeable Haemophilus influenzae* can stimulate mouse epithelial cells to release IL-17C, recruit neutrophils into inflamed tissues, and promote lung tumour growth.^188^ In addition, macrophage infection with *Campylobacter conisus* (*C. conisus*) leads to the upregulation of PRRs and aggregation of interferon-inducible protein 16 inflammasome, which may be a related pro-inflammatory mechanism underlying the ability of *C. conisus* to cause oesophageal cancer.^189^ Moreover, *F. nucleatum*-mediated inhibition of autophagy in colon cancer epithelial cells promotes ROS accumulation, triggering the production of pro-inflammatory cytokines, such as IL-8, IL-1β, and TNF-α.^190^

In addition, microbes can also induce macropinocytosis, an endocytic activity that cells use for antigen capture and delivery, to activate inflammation.^191^ A mouse study found that the Wnt pathway activates macrophage proliferation and stimulates macrophage uptake of bacteria and their products into the colon. However, transcriptional targets for macropinocytosis activation by the Wnt pathway remain unknown; some potential candidates for Wnt-dependent transcription factors involve RAB5, PDK1, and PAK1.^192,193^ The crosstalk between intratumoural microorganisms and polymorphonuclear neutrophils (PMNS) can also promote cancer development. The loss of neutrophils was found to promote the enrichment of *Akkermansia* in a mouse model of CRC. The intratumoural bacteria can boost IL-17 production and intratumoural B cell infiltration, thereby promoting tumour growth and cancer progression.^194^

The inflammatory reaction is accompanied by the immune response. Stress and tissue damage from microbial infection recruit immune cells to promote inflammation and further activate various pro-tumoural inflammatory factors.^195^ The chronic inflammatory microenvironment eventually transforms into an immunosuppressive microenvironment to promote tumour progression and inhibit antitumour immunity.^196^ Moreover, inflammatory cells at sites of infection can also produce ROS to induce DNA damage. The latter can also amplify inflammatory responses, leading to increased DNA damage.^197^ Therefore, crosstalk between various pathogenic mechanisms may contribute to cancer development.

### Immune evasion

The interaction between microorganisms and their host is essential for sustaining immune homoeostasis.^198^ Intratumoural microbes can evade the immune response and affect tumorigenesis by promoting an immunosuppressive microenvironment and immune cell inactivation.

*F. nucleatum* regulates the tumour immune microenvironment by selectively aggregating tumour-infiltrating myeloid cells, including CD11b+ myeloid cells, MDSCs, tumour-associated macrophages, classical myeloid DCs, and CD103+ regulatory DCs, thereby potentiating tumorigenesis.^199^ Besides this, commensal bacteria triggered Myd88-dependent IL-1β and IL-23 production, driving the activation of Vγ6 + Vδ1 + γδ T cells and the subsequent release of IL-17 and other cytotoxic effectors to promote an inflammatory immunosuppressive environment and lung tumour cell proliferation in mouse.^200^ Another mouse study revealed that *Pasteurella* was positively correlated with cytotoxic CD8+ tumour-infiltrating lymphocytes (TILs) and negatively correlated with M2-like macrophages, whereas *Coriobacteriaceae* was positively associated with M2-like macrophages and negatively associated with CD8+ cells. All these immune responses influence the initiation and development of lung tumours.^201^ Certain microorganisms, such as *Staphylococcus aureus*, HBV, and HCV, potentially promote the progression of prostate and liver cancers by promoting the immunosuppression mediated by T regulatory cells (Tregs).^202–204^ Moreover, a large number of Tregs have been observed in HPV-induced cervical lesions, and the level of Tregs is associated with the severity of the disease, suggesting that Tregs may be involved in the interference of anti-HPV immune response.^205^ Furthermore, in both in vivo and in vitro trials, the fungal community of PDAC tissue stimulated the expression of cancer-cell-specific IL-33, which leads to the recruitment and activation of Th2 cells and innate lymphoid cells 2, consequently promoting tumour progression.^206^

As noted previously, intratumoural microbiota can also evade immune responses by promoting the inactivation of immune cells. In a mouse model, the PC microbiome promotes suppressive M1 macrophage differentiation via differentially activating selective TLR to induce T cell anergy.^91^ Elevated lung SCFA levels from lower airway anaerobic bacteria may hinder the production of interferon gamma (IFN-γ) by CD4+ and CD8 + T cells and cause effector T cell depletion, which promotes tumour growth.^207^ HPV downregulates the antigen-presenting pathway through its gene expression programme to inhibit cytotoxic T and NK cell activation, thereby increasing virus replication and transmission and promoting malignant transformation in human cervical cancer cells.^208^ Another in vitro study showed that HPV E7 in cervical cancer directly upregulated intratumoural surface programmed death-ligand 1 (PD-L1) and inhibited cytotoxic T cell function.^209^ Moreover, *F. nucleatum* protein could bind and activate T Cell immunoreceptor with immunoglobulin and immunoreceptor tyrosine-based inhibitory motif domains (TIGIT) and carcinoembryonic antigen cell adhesion molecule 1 (CEACAM1) receptors express on human NK cells and other lymphocytes, inhibiting antitumour immune cell function in CRC.^210,211^ Similarly, the HopQ outer-membrane adhesin of *H. pylori* interacts with CEACAM1 to inactivate immune cells and mediate the migration of VirF cytotoxin-associated gene A (CagA) into host cells and production of IL-8, thereby promoting GC progression.^212,213^ In addition, CagA can stimulate PD-L1 expression in gastric epithelial cells, creating premalignant lesions progressing to GC.^214^ Further studies have shown that the process might be mediated by the Sonic Hedgehog signalling pathway.^215^ EBV infection of B cells and NPC cell lines can induce the downregulation of indoleamine 2, 3-dioxygenase (IDO), resulting in T cell surveillance inactivation.^216,217^

However, many studies have demonstrated that intratumoural microbiota can mediate immune activation and produce antitumour immunity. HPV was associated with massive infiltration of IFN-γ + CD8 + T cells and IL-17 + CD8 + T cells in HNSCC, which might play a key role in the significantly better response to immunotherapy in HPV-positive patients.^218^ The intestinal microbe *Bifidobacterium* has been shown to preferentially colonise in tumour sites and enhance STING/IFN-I signalling in tumour-infiltrating DCs, thereby promoting T cell-dependent antitumour responses in mouse.^31^ Another mouse model of CRC showed that colonisation with *Helicobacter hepaticus* correlates with increased infiltration of CD11c+ myeloid cells, T and B cells, leading to reduced tumour burden.^219^ Moreover, *Lactobacillus plantarum*-derived indole-3-lactic acid increased IL-12a production of DCs by promoting H3K27ac binding in the IL-12a enhancer region, therefore initiating the CD8 + T cell immunity against tumour growth.^220^ Another mouse experiment found that intratumoural *Lactobacillus reuteri* (*Lr*) releases dietary tryptophan catabolite indole-3-aldehyde to promote IFN-γ-producing CD8 + T cells, thereby enhancing immune checkpoint inhibitors (ICIs).^221^ The *Clostridiales-*derived metabolite trimethylamine N-oxide activates the endoplasmic reticulum stress kinase PERK, which causes gasdermin E-mediated pyroptosis in tumour cells and promotes CD8 + T cell-mediated antitumour immunity in BC in vivo.^222^ The metabolite inosine produced by the bacterium *Bifidobacterium pseudolongum* in a mouse model of CRC induces the expression of Th1-regulating genes in CD4 + T cells, thus promoting antitumour immunotherapy.^223^

Overall, microbiota and their derived metabolites are also potential therapeutic targets for supporting immunotherapy, and their effects are context-dependent and require further clarification prior to clinical translation.

### Metabolic regulation

Alterations in human metabolism caused by the microbiome may lead to various metabolic diseases and cancers.^224^ Hieken et al. reported that microbiota in benign breast disease tissue was associated with increased cysteine and methionine metabolism, glycosyltransferases, and fatty acid biosynthesis. By comparison, microbiota in BC tissue, including *Fusobacterium*, *Atopobium*, *Hydrogenophaga*, *Gluconacetobacter*, and *Lactobacillus*, reduce inositol phosphate metabolism.^225^ In cervical cancer, non-*Lactobacillus* dominated communities, comprising *Gardnerella*, *Prevotella*, *Streptococcus*, and *Atopobium*, affected amino acid and nucleotide metabolism.^226^ Dai et al. discovered that the relative level of carbohydrates, carbohydrate conjugates, amino acids, glycerophospholipids, and nucleosides in gastric tumour tissues was higher than that in non-tumour tissues through untargeted metabolomic analysis; subsequent analysis indicated that *Helicobacter* and *Lactobacillus* exhibited negative and positive correlations, respectively, with the majority of differential metabolites in the amino acids, carbohydrates, nucleosides, nucleotides, and glycerophospholipids classes.^227^ Yost et al. showed that the microbiota in patients with OSCC was associated with the upregulation of the activities of iron ion transport-related enzymes, tryptophanase, glutamate dehydrogenase, starch synthase, and superoxide dismutase.^228^ Moreover, Sun et al. found that *F. nucleatum* promoted glucose transporter 1 (GLUT1) upregulation and lactic acid accumulation by triggering GalNAc-Autophagy-TBC1D5 signalling, leading to OSCC progression.^229^ In vitro experiments showed that the expression of Merkel Cell Polyomavirus (MCPyV) oncoprotein can increase the expression of glycolytic genes, including the monocarboxylate lactate transporter SLC16A1 (MCT1), and induce aerobic glycolysis, which is typically characteristic of malignant and rapidly proliferating tumour cells.^230^ KSHV infection has been demonstrated to induce stable glycolysis via increasing HIF1α expression and activating the PI3K/Akt/mTOR signalling pathway.^231–233^ Metabolic flux experiments confirmed that human cytomegalovirus (HCMV) markedly upregulated the glycolytic pathway, tricarboxylic acid cycle, and fatty acid biosynthesis pathway in infected cells.^234^ In addition, HPV E7 protein interacts with and accumulates the dimeric form of M2 type pyruvate kinase (M2PK), a low-activity form that has been found to be upregulated in multiple tumours.^235^ Other cancer-causing viruses, such as EBV, HBV, and HCV, promote cancer by targeting transcription factors, oncogenes, and tumour suppressors to regulate metabolic enzymes and signalling pathways.^236^ However, many non-carcinogenic viral infections have similar metabolic alterations. This suggests that metabolic changes induced by tumour viruses may not be sufficient to cause carcinogenic effects. Nevertheless, in the microenvironment of hypoxia, inflammation and immunosuppression, the presence of pro-tumoural metabolism may potentially promote virus-induced cancerisation.

### Activating invasion and metastasis

Intratumoural microorganisms can alter the internal features of oncocytes and their external microenvironment to promote cancer metastasis.^237^ On the one hand, they directly modulate cancer cells to cope with an unfavourable environment.^238,239^

The epithelial-mesenchymal transition (EMT) process imparts the transition of carcinoma cells with a metastatic mesenchymal phenotype via the TGFβ/SMAD, PI3K/AKT, Wnt/β-catenin, and MAPKs signalling pathways, which drives the invasion and spread of carcinoma cells.^240^ Multiple investigations have demonstrated the association between microorganisms and EMT.^241^ A novel virulence protein of *F. nucleatum*, Fn-Dps, induces EMT by upregulating chemokines CCL2/CCL7, thus promoting the invasion and metastasis of mouse CRC cells.^242^ Li et al. discovered that *E. coli*, *Butyrate-producing bacteria SM4/1*, and a species of *Oscillatoria* were correlated with the production of EMT-related genes in bladder cancer, including E-cadherin, vimentin, SNAI2, SNAI3, and TWIST1.^243^ In an in vitro study, *P. gingivalis* was found to increase the expression of the EMT-associated transcription factors Slug (SNAI2), Snail, and Zeb1 as well as the levels of phosphorylated glycogen synthase kinase-3 beta, an important EMT regulator, thereby promoting the migration of oral epithelial cells.^244^ The presence of *Candida* in advanced metastatic colon tumours might play a role in the downregulation of genes mediating cellular adhesion, including *PTK2B*, *CDKN2C*, and *NET1*, thereby leading to metastasis.^11^ The microbiota can affect EMT and enhance the expression of cancer stem cell (CSC) markers. Bessède et al. reported that infection with *H. Pylori* CagA-positive strain triggers EMT-like changes and high expression of CD44 in vitro, a known gastric CSC marker, leading to an enhanced ability of cells to migrate, invade, and form tumour spheres.^245^ In a mouse experiment, ETBF toxin induces downstream β-catenin nuclear localisation by cleavage of E-cadherin in BC and subsequently enhances stemness potential, tumour growth, and metastatic progression.^246^ *Staphylococcus*, *Lactobacillus*, and *Streptococcus* were highly abundant in a mouse spontaneous breast tumour model and could restrain the RhoAROCK signalling pathway to remodel the actin cell skeleton, thereby improving the resistance of tumour cells to flow shear stress (FSS) and promoting tumour metastasis.^85^ The adhesion of tumour cells to endothelial cells in the bloodstream is another key stage of invasion and metastasis.^247^ *F. nucleatum* mediated a novel PRR, ALPK1, that triggered the NF-κB signalling pathway and upregulated ICAM1, thereby boosting CRC cell adhesion to endothelial cells and metastasis.^248^

Further, the intratumoural microbiota forms a microenvironment conducive to cancer metastasis. In a mouse model of CRC, tumour-resident *E. coli* disrupted the gut vascular barrier through the VirF, promoting the spread of bacteria to the liver and the recruitment of metastatic cells.^249^ Furthermore, *F. nucleatum* colonises BC and suppresses the accumulation of tumour-infiltrating T cells through the abundant Gal-GalNAc on tumour cells, thereby facilitating tumour metastasis in mouse.^33^ Many studies have shown that extracellular vesicles (EVs) trigger pro-inflammatory signalling and activate immunosuppression by modulating communication between cancer cells and their surrounding microenvironment as well as distal organ cells, thereby establishing a pre-metastatic niche for tumour metastasis.^250,251^ A recent work indicated that *F. nucleatum*-derived EVs in BC tissue enhance tumour cell migration and invasion via TLR4.^252^ However, few studies have investigated whether intratumoural microbiota-derived EVs play a key role in promoting metastasis or fully described the mechanisms involved, which may be a worthy direction for future research.

## Prognostic role of intratumoural microbiota

Microbiota composition differs significantly between tumours and healthy tissues, as well as among distinct tumour stages, gene mutations, and distant tumour metastasis, making it a potential prognostic tool.^8^ As our understanding of the influence of intratumoural microorganisms on tumorigenesis deepens, applying these profiles in precision oncology becomes more likely. Here, we briefly summarise the application of various intratumoural microbiota to predict cancer survival and therapeutic efficacy (Table 2).

**Table 2:** Prognostic role of intratumoural microbiota

Cancer types	Pathological type	Stage	Treatment	Microorganism	Outcomes	Survival
**Oesophageal cancer**	Squamous cell carcinoma, adenocarcinoma, and others	**—**	Neoadjuvant chemotherapy/ chemoradiotherapy, surgery	Species *Fusobacterium nucleatum*^253^	Risk factor	OS
	Squamous cell carcinoma	All stages	Neoadjuvant chemotherapy/ chemoradiotherapy, surgery	Species *Fusobacterium nucleatum*^269^	Risk factor	PFS
**Lung cancer**	Non-small cell lung cancer	Stage II	Surgery	Order *Actinomycetales*, order *Pseudomonadales*^261^	Risk factor	DFS
	Non-small cell lung cancer	Stage III, IV	Surgery/ biopsy, targeted therapy/ chemotherapy	Speices *Haemophilus parainfluenzae*^262^	Risk factor	OS
				Species *Serratia marcescens*, species *Acinetobacter jungii*, species *Streptococcus constellation*^262^	Protective factor	OS
	Non-small cell lung cancer	All stages	**—**	Genus Thermus, Genus Legionella^263^	Risk factor	**—**
	Non-small cell lung cancer	Stage IIIB, IV	Chemotherapy, immunotherapy	Species *Helicobacter pylori*^274^	Risk factor	OS, PFS
**Liver cancer**	Hepatocellular carcinoma, intrahepatic cholangiocarcinoma, combined HCC and iCCA	**—**	Surgery	Family *Pseudomonadaceae*^54^	Protective factor	OS
**Colorectal cancer**	**—**	All stages	Surgery	Species *Fusobacterium nucleatum*^254^	Risk factor	OS
	**—**	All stages	Surgery	Genus *Fusobacterium*, genus *Granulicatella*, genus *Gemella*^260^	Protective factor	DFS
	**—**	All stages	Biopsy	Species *Porphyromonas gingivalis*^265^	Risk factor	OS
	**—**	Stage IV	**—**	Genus *Candida*^11^	Protective factor	**—**
				Genus *Saccharomyces*^11^	Risk factor	**—**
**Gastric Cancer**	**—**	All stages	Surgery	Species *Fusobacterium nucleatum*^255^	Risk factor	OS
	**—**	All stages	Surgery, chemotherapy	Species *Helicobacter pylori*^270^	Protective factor	OS
	**—**	**—**	Surgery	Species *Helicobacter pylori*^271^	Protective factor	OS
	**—**	Stage II, III	Surgery, chemotherapy	Species *Helicobacter pylori*^272^	Protective factor	OS, DFS
	Adenocarcinoma, squamous carcinoma, carcinoid carcinoma	Stage III, IV	Immunotherapy	Species *Helicobacter pylori*^275^	Risk factor	PFS
**Pancreatic cancer**	Pancreatic ductal adenocarcinoma	All stages	Surgery, chemotherapy	Genus *Fusobacterium*^257^	Risk factor	OS
	Pancreatic ductal adenocarcinoma	Stage I, II	Surgery, neoadjuvant chemoradiotherapy	Genus *Pseudoxanthomonas*, genus *Streptomyces*, genus *Saccharopolyspora*, genus *Bacillus clausii*^264^	Protective factor	OS
**Melanoma**	**—**	**—**	Immunotherapy	Genus *Clostridium*^7^	Protective factor	**—**
				Species *Gardnerella vaginalis*^7^	Risk factor	**—**
	**—**	Stage III, IV	Immunotherapy	Species *Helicobacter pylori*^273^	Risk factor	OS
	**—**	**—**	Immunotherapy	Genus *Cladosporium*^12^	Risk factor	**—**
**Oral cancer**	Oral squamous cell carcinoma	All stages	Surgery	Species *Fusobacterium nucleatum*^258^	Protective factor	OS
**Anal Squamous Cell Carcinoma**	**—**	All stages	Radiotherapy/chemoradiotherapy, surgery	Species *Fusobacterium nucleatum*^259^	Protective factor	OS
**Vulvar squamous cell carcinoma**	**—**	Stage I, II, and III	Surgery, chemotherapy/ radiotherapy	Species *Fusobacterium nucleatum*, species *Pseudomonas aeruginosa*^256^	Risk factor	PFS
**Ovarian cancer**	**—**	**—**	Surgery	Genus *Phaeosphaeriaceae*^12^	Risk factor	PFS
**Kidney cancer**	**—**	**—**	**—**	Human endogenous retrovirus^118^	Risk factor	OS

The abundance of *F. nucleatum* is correlated with the short survival of many cancers.^253–255^ *F. nucleatum* and *Pseudomonas aeruginosa* have been identified as tumour-promoting bacteria linked to poor outcomes in vulvar squamous cell carcinoma patients.^256^ The colonisation of *Fusobacterium* species in patients with PDAC is also associated with short survival.^257^ However, in some cases, *F. nucleatum* was unexpectedly found to be positively correlated with survival, such as in OSCC^258^ and anal squamous cell carcinoma.^259^ Moreover, Alexander et al. recently found that a cluster of microbiota that included *Fusobacterium*, *Granulicatella*, and *Gemella* independently predicted higher disease-free survival (DFS) in patients after CRC resection,^260^ despite previous studies linking high *F. nucleatum* abundance to poor prognosis of CRC.^254^ This paradox may be due to the enhanced immune response triggered by an increased abundance of the microbial cluster, potentially leading to persistent immune memory against CRC post-surgery and vigilant monitoring for recurrence and metastasis. Therefore, the contribution of microorganisms to prognosis should be considered in the context of different organ tumours and treatment modalities.

In addition to *F. nucleatum*, other microbiota associations with prognosis have been identified. For instance, a higher burden of the *Actinomycetales* and *Pseudomonadales* orders was related to lower DFS in stage II non-small cell lung cancer (NSCLC) tumour tissue.^261^ Analysis of the microbiota in first-line treatment of NSCLC samples revealed that the prevalence of certain bacteria, including *Haemophilus parainfluenzae*, *Serratia marcescens*, *Acinetobacter jungii*, and *Streptococcus constellation*, effectively predicted 2-year survival.^262^ Moreover, *Thermus* is more prevalent in advanced-stage patients, whereas *Legionella* is more abundant in metastatic patients.^263^ Conversely, the abundance of *Pseudomonadaceae*, which have antitumour effects, was reduced in tumour tissues and linearly related to the prognosis of PLC patients.^54^ Riquelme et al. discovered that the intratumoural microbiota, including *Pseudoxanthomonas*, *Streptomyces*, *Saccharopolyspora*, and *Bacillus clausii*, could be utilised as an ideal combination to predict the long-term survival of PC.^264^ Patients with *P. gingivalis* infection were found to exhibit substantial reduction in cancer-specific survival in CRC.^265^ The *Candida*-to-*Saccharomyces* ratio was positively correlated with the stage of CRC.^11^ In ovarian cancer, patients with intratumoural *Phaeosphaeriaceae*, or related *Phaeosphaeria* genus, had substantially decreased progression-free survival (PFS).^12^ In addition, high HERV expression in kidney cancer was associated with poorer survival.^118^ A recent prospective analysis demonstrated the prognostic significance of the measurable microbiome in soft tissue sarcoma, especially intratumoural viral microbiome, which is associated with higher NK cell infiltration and improved clinical outcomes.^266^ Beyond the individual roles of certain microorganisms, microbial clustering or combinations demonstrate unique prognostic value. Sun et al. identified two hepatotypes based on the microbial profile clustering, representing independent prognostic factors in patients with resected HCC.^267^ Song et al. developed a microbiome-related score model based on 27 microorganisms that acted as an independent prognostic factor for HCC patients.^268^

Specific intracellular microorganisms have also been related to immunotherapy efficacy across various cancers. A higher abundance of *F. nucleatum* is correlated with the reduced efficacy of neoadjuvant chemotherapy in oesophageal squamous cell carcinoma. Targeting this bacterium with antibiotics may potentially enhance the curative effect in oesophageal squamous cell carcinoma patients.^269^ In patients with metastatic melanoma receiving immunotherapy, *Clostridium* is enriched in tumours that responded to ICIs, while *Gardnerella vaginalis* is enriched in the tumours of non-responders.^7^ Interestingly, previous research suggested that *H. pylori* infection is correlated with a favourable outcome in GC patients.^270–272^ However, recent studies have indicated that the presence of *H. pylori* may affect the efficacy of ICIs in melanoma,^273^ NSCLC,^274^ and advanced GC,^275^ introducing a new paradox regarding the varied effects of the same microorganism on cancer occurrence, prognosis, and treatment efficacy. Fungi also play a role in immunotherapy response in metastatic melanoma, with the *Cladosporium* genus significantly enriched in non-responders.^12^ Therefore, further understanding of whether intracellular microbiota could interfere with drug efficacy is essential as well as exploring whether targeted microbiota therapy represents an effective strategy to improve tumour efficacy and patient prognosis.

Prospective microbiome studies have aided in cancer diagnosis and prognosis. Although the microbiome as a prognostic factor requires further research and validation, if its predictive and prognostic capabilities are confirmed, this biomarker could significantly advance the goal of precision medicine for patients with cancer. Unlike intestinal and blood microbiota, intratumoural microbiota is in close proximity to tumour cells, allowing for a more accurate reflection of the actual state of the tumour. However, given that obtaining tissue samples are invasive and difficult, many current studies often use alternative specimens, such as sputum, BALF, and airway brushing tissue, to screen for lung cancer; however, they may introduce certain errors. With the rapid advances in technology and bioinformatics approaches, combining microbiome-based blood diagnostics and imaging evidence of their spatial distribution may improve cancer detection and prognosis. Furthermore, integrating multiple-omics data through artificial intelligence with other emerging sampling techniques, such as ingestible capsules for microbiome sampling, will enable further precision diagnosis and prognostic strategies.^276–278^

Nevertheless, challenges still exist regarding environmental pollution, low microbial biomass, and antibiotic perturbation. Moreover, most research has focused on advanced tumours, highlighting the need for future work to focus on detecting precancerous lesions or early-stage tumours to achieve early diagnosis and treatment.

## Applications of intratumoural microbiota in cancer therapy

Rapid identification and treatment achieve better outcomes in patients with cancer; treatment strategies vary depending on the type of cancer. Different cancer subtypes, mutation states, and degrees of invasion need specific treatment for optimal efficacy, making individualised treatment particularly important.^279^ Some specific microorganisms have been reported as direct factors responsible for the development of cancer. Certain medications have been used clinically to eradicate microorganisms to prevent cancer as much as possible and to assist in the treatment of cancer; these include the use of quadruple antibiotics to prevent and treat *H. pylori*-derived GC,^280–282^ direct antiviral drugs (DAAs) against HCV,^283,284^ as well as vaccination against HPV and HBV to prevent cervical, head-and-neck, and liver cancer.^285,286^ Due to the significance of the microbiome in carcinogenesis, targeting intratumoural pathogenic microbiota may be beneficial for precise cancer therapy and prevention of recurrence.

### Antibiotics

**Fig. 4:**
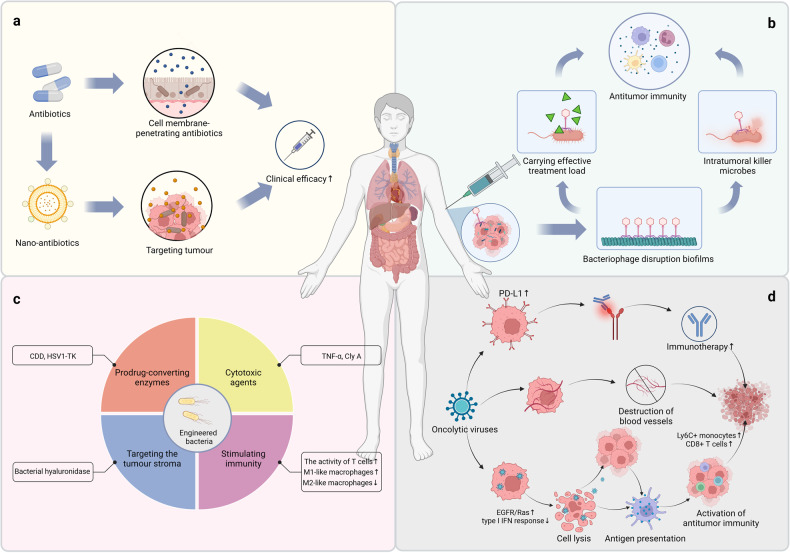
**Application of intratumoural microbiota in cancer therapy**. **a** Antibiotics. Cell membrane-penetrating antibiotics and nano-antibiotics can target intratumoural microorganisms, thereby improving clinical drug efficacy. **b** Bacteriophages. Bacteriophages can precisely target and eliminate harmful microbes within tumours and provide an effective therapeutic load to attract antitumour immune cells to the attack site by being modified into programmable bacterial assassins. **c** Engineered bacteria. Engineered bacteria can exert significant antitumour effects through a variety of payload delivery and effector systems, such as the production of prodrug-converting enzymes, expression of controlled cytotoxic agents, stimulation of immune responses, and targeting of tumour stroma. **d** Oncolytic viruses. Oncolytic viruses can exploit dysregulated signalling pathways to cause cell lysis and death or damage blood vessels to reduce tumour cell growth. Oncolytic viruses can also increase PD-L1 expression in tumours and immune cells, resulting in more sensitive targets for anti-PD-L1 immunotherapy. Created with BioRender.com

*Gamma-Proteobacteria* present in PDAC produces the bacterial enzyme cytidine deaminase (CDD) to metabolise the chemotherapy drug gemcitabine (2’, 2’-difluorodeoxycytidine) into its ineffective form (2’, 2’-difluorodeoxyuridine), thereby reducing its efficacy and leading to drug resistance in PC.^90^ *F. nucleatum* is abundant in CRC chemotherapy patients’ tissues, reduces CRC apoptosis, and causes CRC resistance to Oxaliplatin and 5-fluorouracil.^287^ Multiple retrospective investigations have found that antibiotic therapy improves cancer patients’ survival (Fig. 4a). For example, patients treated with gemcitabine-based and 5-FU-based chemotherapy respectively, were found to have significantly prolonged PFS after antibiotic therapy.^288^ In addition, metronidazole reduced the amount of *Fusobacterium*, reproduction of carcinoma cells, and development of tumours in a mouse model of CRC.^289^ A recent study found that aerosolised and oral absorbable antibiotics can reduce mouse mammary tumour growth, and it also showed that paclitaxel treatment in combination with ampicillin improved the chemotherapeutic efficacy in BC.^290^ Another mouse study indicated that antibiotics could counteract the metastatic effects of *F. nucleatum* in BC.^33^

However, the systemic administration of antibiotics can not only eliminate the tumour microbiota but also disturb the gut microbiome, which further affects the efficacy of antitumour therapy. As the intestinal microbiota plays a crucial role in tumour development, the consequences of antibiotic use via different administration routes can be complicated.^85^ A study demonstrated that exposure to antibiotics shortly before or after the start of ICI could lead to the disruption of microbiota, which may be detrimental to ICI efficacy.^291^ Moreover, Jing et al. found that antibiotic treatment was linked with an increased risk of immune-related adverse events among cancer patients receiving anti-programmed cell death protein 1 (PD-1)/PD-L1 therapies.^292^ Stein-Thoeringer et al. recently demonstrated that treatment with wide-spectrum antibiotics before CD19-targeted chimeric antigen receptor (CAR)-T cell therapy is associated with adverse outcomes.^293^ Additionally, Laura et al.’s study indicated that gut dysbiosis induced by the wide-spectrum antibiotic cocktail treatment in mouse epithelial ovarian cancer could promote tumour growth and suppress cisplatin sensitivity.^294^ Although the advantages and disadvantages of implementing antibiotics in cancer treatment are still difficult to clarify due to their intricate effects on gut microbiota, clearance of the tumour-infiltrating microbiome (TIM) is a key point in cancer therapy. Thus, further exploration is required into precisely targeting TIM without disrupting the gut microbiome. Administration of the cell membrane-penetrating antibiotics, such as doxycycline, can be a potential choice.^85^ Gao et al. developed metronidazole-fluorouridine nanoparticles (MTI-FDU) in a hydrophilic solution, and they achieved TIM targeting through increasing penetrability and retention effect in solid tumours.^295^ Further in-depth analysis of how to efficiently eliminate the pro-tumoural TIM and keep a balanced microbiota system will provide more insights into cancer therapies.

### Bacteriophages

The dysbiotic influence of antibiotics on microorganisms, bacterial resistance, and the impenetrability of biofilms have made it challenging to accurately ablate the microbiome in tumours using antibiotics.^296–299^ However, bacteriophages can precisely target and eliminate harmful microbes within tumours,^300^ and several *Fusobacterium*-targeted phages were shown to effectively invade bacteria within tumours (Fig. 4b). Researchers are now utilising synthetic biology techniques to modify phages into programmable bacterial assassins capable of delivering an effective therapeutic load to attract antitumour immune cells to the attack site.^301^ For example, azide-modified phages targeting *F. nucleatum* have been studied to covalently bind irinotecan nanoparticles and improve their delivery to mouse colon tumours.^302^ Although many studies have reported the safety of bacteriophages because they only reproduce in bacteria, they have been shown to induce host inflammation and immune responses.^303,304^ Therefore, determining the type, dosage, and mode of administration of bacteriophages is essential.^305^

Specificity, one of the advantages of bacteriophages as a customised fungicide, instead limits the therapeutic efficacy of bacteriophages by restricting their ability to kill only a specific type of bacteria. In this case, therapeutic bacteriophages must be specially selected and produced according to the different bacterial strains of different patients. Expanding the bactericidal range of bacteriophages is the focus of current research. Of those, cocktail therapy with a mixture of multiple bacteriophages is currently considered the best bacteriophage regimen.^306^ Bacteriophages are more suitable as a complement for antibiotics or a last resort when other effective therapies are not available; however, they are not a complete substitute for antibiotics.^307^

### Engineered bacteria

Given the advent of synthetic biology, multiple bacteria have been modified to have lower toxicity and higher capability to accurately target tumour tissues.^308^ These tumour-targeting bacteria play a significant antitumour role via a variety of payload delivery and effector systems, such as the production of prodrug-converting enzymes, expression of controlled cytotoxic agents, stimulation of the immune response, and targeting the tumour stroma (Fig. 4c).^309^

Some bacteria produce prodrug-converting enzymes that enhance drug efficacy, including CDD. A study discovered that intratumoural injection of attenuated *Salmonella typhimurium* (*S. typhimurium*) strains VNP20009 expressing *E. coli* CDD significantly increased intratumoural 5-FU levels in cancer patients.^310^ The *Bifidobacterium infantis*-mediated prodrug enzyme delivery system of herpes simplex virus type I thymidine kinase/ganciclovir (HSV1-TK/GCV) significantly reduced bladder cancer progression in a rat model.^311^ Moreover, tumour-targeting bacteria can be engineered to express controlled cytotoxic drugs with antitumour effects. Utilising tumour-targeting systems with the non-pathogenic *E. coli* strain, MG1655 induced targeted production of TNF-α in mouse tumours, which demonstrated the tumour-suppressive effect.^312^ In two other mouse studies, the expression of cytolysin A (Cly A) in *E. coli* or *S. typhimurium* was regulated by inducible or constitutive promoters, achieving localisation to tumour tissue.^313,314^ Engineered bacteria can enhance antitumour activity by stimulating immune responses. A probiotic strain of *E. coli* Nissle 1917 was engineered to target mouse tumours and transform the accumulated metabolic waste product, ammonia, in the TME into L-arginine, which increased T cell activity and enhanced the efficacy of immunotherapy.^315^ Moreover, researchers designed an attenuated *S. typhimurium* strain that released *Vibrio trauma* flagellum B (FlaB) in the tumour tissue of the mouse and caused M1-like macrophages to increase and M2-like macrophages to decrease through TLR4 signalling.^316^ In addition, *Listeria* harbouring-galactosylceramide could enhance the activity of NKT cells and further reduce metastasis in a mouse breast tumour model.^317^ By taking advantage of bacterial enzymes targeting the tumour stroma, engineered bacteria can also be applied to treat solid tumours that are difficult to target using conventional therapies. Ebelt et al. engineered an attenuated tumour-targeting strain of *S. typhimurium* secreting functional bacterial hyaluronidase that degraded the deposition of human hyaluronic acid and significantly enhanced penetration of chemotherapeutic agents in orthotopic human PDAC mouse models.^318^ Moreover, many natural bacteria have antitumour effects, such as *Staphylococcus epidermidis*, which is capable of producing 6-N-hydroxy-aminopurine, a molecule that inhibits DNA polymerase activity to selectively inhibit the proliferation and development of skin tumours in mouse.^319^ Modifying these natural bacteria to target tumour inhibition will be a new research prospect.

Bacteria-based tumour therapy could provide multiple opportunities, but most research is still in the preclinical phase. This may be due to underlying differences between model organisms and humans in their genetic backgrounds or disease heterogeneity. Moreover, safety issues cannot be ignored. First, as the pharmacokinetics and dose responses of live bacteria differ from the norm, it poses a challenge in determining the optimal initial dose and schedule of administration. Secondly, engineered bacterial therapy involves transforming tumour foci into locally destructive tumour infections that, if not managed properly, can result in life-threatening infections or sepsis. Third, it is essential to prevent the contamination of the adjacent medical environment by living bacteria.

### Oncolytic viruses

Oncolytic virus (OV) therapy involves intratumoural injection of genetically modified and attenuated viruses to selectively target cancer cells. OVs utilise the susceptibility of tumour cells to viral infection and exploit dysregulated signalling pathways to cause cell lysis and death. The antigens secreted from tumour cell lysis are taken up by antigen-presenting cells to further stimulate antitumour immunity. Moreover, OV can damage blood vessels, thus reducing tumour cell growth (Fig. 4d).^320^

Both in vivo and in vitro studies have demonstrated that the oncolytic poxvirus JX-594 replicates within and attacks tumour cells of various types of cancer by activating the EGFR/Ras signalling pathway and deactivating the type I IFN response pathway.^321^ Moreover, the arenavirus lymphocytic choriomeningitis virus replicates within tumour cells in multiple mouse and human cancers, inducing antitumour immunity by enlisting IFN-producing Ly6C+ monocytes and increasing the abundance of tumour-specific CD8 + T cells.^322^ Chen et al. found that variant OVs generating IL-23 increased the level of antitumour factors and infiltration of activated T cells in the TME, thereby converting the immunosuppressive phenotype and eliciting antitumour immunity in multiple mouse tumour models.^323^ In addition, OVs enhance PD-L1 expression in tumours and immune cells, resulting in more sensitive anti-PD-L1 immunotherapy targets. Coxsackievirus A21 combined with pembrolizumab partially increased the number of PD-L1+ tumour cells and exhibited well tolerance in a clinical trial.^324^ This indicates that the combination of OVs and ICIs may enhance antitumour efficacy while limiting toxicity, compared with that of monotherapy.^325^ Thus far, four OVs have been approved for cancer treatment worldwide, among which Talimogene laherparepvec (T-VEC) for melanoma is the first widely approved and recognised OV.^326^

However, the clinical transformation of OVs remains challenging. Combining OVs and radiotherapy, chemotherapy, or immunotherapy generally achieves a better therapeutic effect than the modalities alone. As replicable live viruses, OVs have received considerable attention in terms of safety. It is necessary to carefully examine the administration method, dosage, volume, time, and drug preservation method. Although intratumoural injection is the main administration route of the OVs,^326^ growing evidence has supported the possibility of intravenous injection.^327^ This possibility raises new problems; for example, OVs may be recognised and eliminated by the immune system, which would significantly decrease their efficacy. Therefore, new delivery methods for OVs have been in development, such as polymer coating or cell vectors.^328–330^ In addition, no reliable predictive biomarkers are available to identify which patients are candidates for OV treatment. Song et al. found that matrix remoulding associated 8 (MXRA8), which is widely and highly expressed in multiple solid tumours, is the receptor and therapeutic biomarker of oncolytic virus M1 (OVM). Further research showed that the tumour selectivity of OVM mainly hinges upon the combination of MXRA8 and zinc-finger antiviral protein.^331^ More similar studies are needed to provide precision medicine guidance for OV treatment.

### Microbial intervention in the context of immunotherapy

Cancer immunotherapy has emerged as a significant means of combating cancer, mainly including immune checkpoint therapy represented by CTLA-4 and PD-1 and adoptive T cell therapy represented by CAR-T therapy.^332^ Based on the established role of host microbiota in modulating immunotherapy responses, many studies have begun to attempt to manipulate the microbiome for therapeutic purposes; faecal microflora transplantation (FMT), probiotics, and antibiotics are examples of this approach.

Vetizou et al. revealed the correlation between *Bacteroides fragilis* (*B. fragilis*) and the efficacy of CTLA-4 blocking in mouse models and melanoma patients. Oral gavage (OG) with *B. fragilis* and FMT from responding humans to mouse altered the immunotherapy response of germ-free, non-responding mouse.^333^ In addition, through FMT from JAX mouse that were effective for anti-PD-L1 therapy to TAC mouse, Sivan et al. showed that PD-L1 antibody levels were enhanced to inhibit melanoma in TAC mouse and further identified *Bifidobacterium* to be the responsible microbe.^334^ Other studies have reported similar results that microbial intervention boosts immunotherapy.^223,335^

These results indicate the benefits of microbial-based interventions in the context of immunotherapy; nevertheless, further studies in patients with different cancer types are warranted. Given the feasibility of stool collection from human donors, the use of FMT in immunotherapy is undoubtedly a promising area. Clinical trials have shown that FMT can benefit melanoma patients.^336,337^ In addition, immunotherapy combined with probiotic administration is another powerful research direction, which is more targeted to combine one or multiple microorganisms into a single formula.^338^ It is worth noting that there are few studies on whether bacteriophages affect immunotherapy responses in cancer patients. A study has shown that a bacteriophage-specific T cell epitope is correlated with anti-PD-1 therapy response in mouse.^339^ Accordingly, screening for differential bacteriophages from patients with different immune response states and combined immunotherapy through an approach similar to probiotic supplementation may help maximise benefits.

However, integrating animal model data into complex human tumours and antitumour immunotherapy is challenging. As mentioned earlier, broad-spectrum antibiotics disrupt the gut microbiota and impair immunotherapy efficacy and long-term patient survival. How to use specific antibiotics to remove immunosuppressive microbes is a key question. In addition, studies have found that non-targeted use of commercially available probiotics may not be beneficial to immunotherapy efficacy and may cause immunotherapy-related autoimmune events.^340–342^ Therefore, additional research is required to identify the underlying mechanisms of these microbial interventions and to develop personalised beneficial probiotics for patients with different living environments and dietary habits.^343^ Meanwhile, in order to avoid the risk of infection in cancer patients with weakened immune systems, beneficial substances produced by microorganisms or prebiotics can be used instead of probiotics.^344^

In addition, most current studies are based on gut microbes, and there are few studies on the role of tumour microbes in immunotherapy efficacy. It remains unknown whether there is a crosstalk between the gut microbes and the intratumoural microbes and whether changing the gut microbes will affect the intratumoural microbes and the characteristics of the host immune microenvironment; these aspects need to be explored. The study by Riquelme et al. may provide a way forward.^264^ Using human-mouse FMT from short- and long-term survival or control donors, they found that gut microbes can differentially regulate pancreatic tumour microbiome, thereby affecting tumour growth and tumour immune infiltration. Moreover, a recent study showed that OG probiotic *Lr* translocated from the gut and persistently colonised the tumours in melanoma mice, promoting the response of CD8 + T cells in TME and enhancing the therapeutic effect of anti-PD-L1.^221^ Thus, these microbial interventions may function through the gut-tumour microbiome crosstalk or a suitable niche provided by the TME, and direct intratumoural targeting may further enhance immunotherapy efficacy in the future. In addition, the research progress of CAR-T therapy for solid tumours is limited, so there are few studies on the influence of intratumoural microorganisms on CAR-T therapy. It is not yet known whether microorganisms can be a new breakthrough for CAR-T treatment of solid tumours.

## Conclusion and outstanding questions

The microbiota has received widespread attention as a critical component in tumours. TME microbiota that modulates tumour development and affects cancer therapy is drawing increasing attention. Multiple tumours are thought to harbour distinct microbial communities, and the intratumoural microbiota may facilitate cancer development through various mechanisms, such as genomic instability and mutation, epigenetic modification, tumour-promoting inflammation, immune evasion, metabolic regulation, and activation of invasion and metastasis. Understanding this complex relationship between microbes and tumours could provide valuable insights into potential and existing cancer treatment options. Many bacteria that have been modified to be less virulent and accurately target tumour tissues have shown significant antitumour effects in preclinical studies. Moreover, microbial combinations with chemotherapy/immunotherapy may reduce drug resistance and improve anticancer efficacy.

Several studies have reported contradictory conclusions when describing the relationship between microorganisms and tumours.^60,61,110–112^ Therefore, standardised protocols for the study of intratumoural microorganisms should be developed and generally adopted. In addition, most studies are microbe-based cross-sectional studies that could not determine a causal relationship between intratumoural microorganisms and tumorigenesis. Although many studies have suggested that intratumoural microbes are the cause of cancer, we cannot deny the fact that microbial changes are the result of the development of certain cancers. Nevertheless, it is meaningful to use longitudinal prospective studies with large sample sizes and further explore the effects of the intratumoural microbiota on cancer cells of different phenotypes as well as immune cells. Recently, Bullman proposed that cancer cells and intratumoural microbiota may have a mutualistic relationship; that is, they both need to evade the immune system, and both have the capability to migrate or spread to new permitted niches.^345^ Therefore, despite the complex crosstalk between microbiota and tumours, it may be possible to treat tumours by modifying or manipulating the intratumoural microbiota.

The study of intratumoural microbiota in tumorigenesis and progression is just the beginning. Sophisticated animal models are needed in the future to trace tumour cells affected by microorganisms to provide more preclinical evidence. Interdisciplinary approaches are also necessary to quantitatively understand the relationship between microbes in tumours and tumour formation and development. Future work should also focus on the relationship between intratumoural microbes and other clinical factors known to be linked to the risk of various cancers, such as inflammatory and metabolic markers, to determine whether these factors have an additional effect on the composition of intratumoural microbiota. In addition, linking tissue microbial differences between healthy and high-risk individuals with cancer development might contribute to establishing more effective cancer prevention and diagnosis methods; however, the effort is complicated by the ethical and accessibility challenges associated with normal human tissue. In terms of antitumour therapy, studies linking identified microbial signatures to tumour response regulation could identify new targets for clinical intervention. Exploring combination therapy strategies based on microbial intervention to improve the clinical treatment effect is another promising research direction.
